# Effect of maltitol-containing chewing gum use on the composition of dental plaque microbiota in subjects with active dental caries

**DOI:** 10.1080/20002297.2017.1374152

**Published:** 2017-09-18

**Authors:** Erica M. Prosdocimi, James O. Kistler, Rebecca Moazzez, Clementine Thabuis, Caroline Perreau, William G. Wade

**Affiliations:** ^a^ Centre for Immunobiology, Blizard Institute, Barts and The London School of Medicine and Dentistry, Queen Mary University of London, London, UK; ^b^ Division of Mucosal and Salivary Biology, King’s College London Dental Institute, London, UK; ^c^ Roquette, Lestrem, France

**Keywords:** Caries, microbiome, maltitol, plaque, chewing gum

## Abstract

***Background*:** Sugar alcohols such as xylitol are incorporated in a number of oral hygiene products for their anti-cariogenic properties while chewing gum is known to be beneficial to oral hygiene.

***Objective*:** The aim of this study was to determine the composition of the dental plaque microbiota in patients with active caries before and after using a chewing gum supplemented with maltitol.

***Design***: Forty subjects with active caries were randomly allocated to chew maltitol gum or gum base for two weeks. A healthy control group used gum base for two weeks. Plaque samples were collected before and after treatment and the microbiota analysed by pyrosequencing of 16S rRNA genes.

***Results***: A total of 773,547 sequences were obtained from 117 samples. There was no difference in structure of the bacterial communities between groups (AMOVA). There was a significant difference in community membership between groups, (AMOVA, p=0.009). There was a significant difference between the control group after treatment and the maltitol patient group after treatment (p<0.001). *A. naeslundii* HOT-176 and *Actinomyces* HOT-169 were significantly reduced following use of maltitol chewing gum in patients.

***Conclusions***: This study has shown that chewing gum containing maltitol had minor effects on the composition of the plaque microbiome.

## Introduction

Dental caries is primarily caused by excessive and frequent consumption of readily fermentable carbohydrates [1]. Bacteria in dental plaque produce acids that degrade the tooth tissues and the local reduction of pH leads to the selection of an aciduric microbiota, which contributes further to lesion development. Polyols such as xylitol can inhibit acid production by plaque, reduce the numbers of acid-producing bacteria, and have been shown to be anti-cariogenic [2]. Chewing gum stimulates salivary flow, promoting the removal of dietary fermentable carbohydrate from the mouth while also removing plaque from the teeth [3]. Polyols can be incorporated into chewing gum to further enhance its anti-plaque and anti-caries properties. For example, maltitol- and xylitol-containing chewing gums significantly reduced gingivitis scores compared with a placebo gum in individuals who refrained from brushing [4]. They were also shown to increase plaque pH and reduce plaque scores and levels of caries-associated bacterial species from the genera *Streptococcus*, *Lactobacillus*, and *Actinomyces* [5]. In spite of belonging to the same chemical family, polyols exhibit differing properties linked to their size, molecular weight, and hydrophobicity. For example, mannitol is highly osmotic, which causes adverse effects on intestinal transit at levels above 10 g/day, while maltitol has excellent digestive tolerance up to 50 g/d. The ability of xylitol to interfere with bacterial metabolism has been well studied, but less is known about maltitol.

Recent improvements in DNA sequencing technologies have made it possible to perform comprehensive, culture-independent analyses of all the bacteria in the human mouth, the oral microbiome. The aim of this study was to investigate the interaction between maltitol and oral bacteria by comparing the composition of the dental plaque microbiota in patients with active caries before and after using a chewing gum supplemented with maltitol or base gum for 2 weeks. Samples were also collected from a healthy control group who used the gum base in order to compare the microbiota at baseline between healthy and carious phenotypes and to determine the influence of mastication in carious and healthy subjects who used the gum base. The composition of the plaque microbiota was determined using a high-throughput culture-independent method targeting the small subunit ribosomal RNA gene.

## Methods and materials

A single-centre, parallel study was performed. Ethical approval for the study was given by the HSC RECA committee, ref. 14/NI/1107. Forty-three patients with active caries were recruited from those attending the undergraduate student clinics and consultant clinics at Guy’s Dental Hospital. Patients were aged ≥18 years and at high risk of dental caries, on the basis of the presence of one or more of the following disease indicators: visible cavities or radiographic evidence of carious lesion penetration into dentine; interproximal radiographic evidence of lesion penetration into dentine; white spot lesions on smooth surfaces; restorations within previous 3 years due to caries. Patients were diagnosed with active dental caries, with a minimum of one active primary lesion in enamel and/or dentine excluding recurrent caries underneath restorations or arrested caries. Treatment of the carious lesions was carried out after the completion of the study. The study was of short duration and was performed while patients were waiting for a treatment appointment.

Twenty healthy volunteers were recruited from staff and students at King’s College London and had no active caries. All subjects had a minimum of 20 natural teeth with no more than four crowns (excluding third molars) present, were in good general health, and had no known history of reactions to sugar alcohols.

Potential subjects were excluded from the study if they had advanced periodontal disease; diseases of the oral soft tissues; orthodontic appliances; abnormal salivary function in conditions resulting in dry mouth, such as Sjögren’s syndrome; had received antibiotics 1 month before or during this study; were pregnant or breastfeeding; had participated in another clinical study in the month preceding this study; were immunocompromised (e.g. suffering from HIV/AIDS or receiving immunosuppressive drug therapy); or regularly consumed more than three sticks of chewing gum per day.

Following recruitment, the subjects were given a standard fluoride toothpaste and tooth brush to use for a minimum of 2 weeks prior to the start of the study, continuing to the end of the study. No additional oral hygiene instruction was given. Subjects were advised to brush in their normal way but use only the products provided. During this period, subjects were asked to refrain from other oral hygiene procedures such as interdental cleaning and not to use any other toothpastes, mouth rinses, chewing gum or sugar-free confectionery. Subjects were randomly allocated to the active chewing gum or control group. Samples of plaque were collected at baseline, following which subjects were asked to use the chewing gum five times (two pieces of SweetPearl® maltitol gum or one piece of gum base) a day for 2 weeks, after which further plaque samples were collected.

The chewing gums were experimental formulations. The maltitol gum was made up of 0.35 g gum base (Cafosa SA, Barcelona, Spain), 0.49 g maltitol (SweetPearl®, Roquette Frères, Lestrem, France), 0.02 g maltitol syrup (LYCASIN HBC 80/55, Roquette Frères), 0.004 g glycerine (Dow Chemicals Company, Midland, MI), and 0.009 g spearmint flavour (Mane S.A., Le Bar-sur-Loup, France). The gum base control was made up of 0.7 g gum base (Cafosa SA), 0.24 g talc (Talc de Luzenac, Rio Tinto Minerals-Luzenac Operations, Luzenac, France), and 0.03 g spearmint flavour (Mane SA). Since the maltitol included more ingredients by weight than the gum base, the formulations were adjusted so that the amount of gum base per intake was the same for both products. One gum base pellet contained 0.7 g of gum base, whereas one pellet of maltitol gum contained 0.35 g of gum base. Consequently, subjects in the maltitol group chewed two pieces each time, whereas those in the gum base control group chewed only one. Subjects gave verbal confirmation of compliance with the chewing regimen and returned the empty chewing gum pots after treatment.

A plaque sample was collected after 2 weeks’ use of a standard toothpaste. Plaque was collected at least 1 h after the last meal from the interproximal areas on all upper and lower premolar and molar teeth. A periodontal probe was passed through the mesial and distal surfaces of each of the teeth. The plaque from each subject at each visit was pooled and placed in 1 mL SSDE buffer (saline-saturated 20% DMSO-0.25M EDTA, pH 8.0) and stored at −70°C until being subjected to analysis.

DNA was extracted from the plaque samples by means of the Genelute DNA extraction kit (Merck, Gillingham, UK) with the additional pre-treatment with lysozyme (Merck) recommended by the manufacturers for Gram-positive bacteria and 16S rRNA genes amplified by PCR with primers 27F (with the YM modification) and 519R, targeting variable regions 1–3. Each sample was amplified using primers incorporating a unique barcode and Roche 454 adapters. PCR amplicons were purified, sized, quantified, and pooled in equimolar proportions. Emulsion PCR and unidirectional sequencing of the libraries were performed using the Lib-L kit and Roche 454 GS-FLX Titanium sequencer at the Department of Biochemistry, University of Cambridge. Processing and analysis of sequences was carried out using the mothur analysis suite version 1.36.1 [6] based on the Schloss SOP (January 2016) [7]. Sequences were clustered into operational taxonomic units (OTUs) at a sequence dissimilarity distance of 0.015 using an average neighbour algorithm and then classified using a naïve Bayesian classifier implemented in mothur with the Human Oral Microbiome Database (HOMD) reference dataset (version 13.2). The α-diversity of bacterial communities based on OTUs were analysed using approaches implemented by mothur: diversity of the communities were estimated using Simpson’s inverse diversity index [8] and the total richness of the communities was estimated using Chao1 [9]. For β-diversity analyses, the datasets were subsampled so that the number of sequences was equal in all samples (equal to that of the sample with the lowest number of sequences in the dataset). To compare the β-diversity of samples based on OTUs, the Jaccard Index (ratio of shared OTUs to distinct OTUs) and the θ-YC metric (compares community structure by accounting for the relative abundance of taxa) [10] were used to generate distance matrices in mothur, and visualized as ordination plots by nonmetric multidimensional scaling (NMDS) plots. Ordination plots were generated in R (r-project.org) using the rgl and ggplot2 packages.

Analysis of molecular variance (AMOVA) [11] as implemented by mothur was performed to determine whether clustering patterns seen in the NMDS plots were statistically supported by differences in the distance matrix. Homogeneity of molecular variance (HOMOVA) was performed to compare the genetic diversity between groups. Parsimony [12] was used to compare community structure between groups. Samples were compared between treatment groups and over time within groups.

An oligotyping analysis was also performed. Sequences identified as belonging to the genus *Actinomyces* were extracted from the quality-filtered mothur dataset and formatted using the ‘mothur2oligo’ tool (available at https://github.com/michberr/MicrobeMiseq/tree/master/mothur2oligo). Minimum entropy decomposition (MED) analysis [13] was performed on 33,777 reads from 117 samples with MED pipeline version 2.1 (available at https://meren.github.io/projects/med/). The parameters used were minimum substantive abundance of a MED node (-M) = 7 and maximum variation allowed in each node (-V) = 4 nt. The MED algorithm is similar to the previously described oligotyping algorithm [14], differentiating taxa on the basis of single-nucleotide differences in the positions of highest entropy. This method results in a very fine discrimination of taxa and deals effectively with sequencing errors. Compared with oligotyping, the MED pipeline allows an automated analysis, which, needing less supervision from the user, can be applied to taxonomic groups of higher diversity with fast and reproducible outputs.

To identify the MED nodes at species level, a representative sequence from each was compared to the HOMD database through the web tool (available at http://www.homd.org).

The matrix describing the number of sequences of each MED node present in each sample was first converted to a proportion matrix dividing each count by the total number of sequences for that sample. Counts of oligotypes from the same species were combined to give species-level relative abundances. These were then compared before and after treatment using the paired Wilcoxon test.

## Results

Forty-three patients with active caries and 20 control subjects without caries were consented and entered into the study. Three subjects were withdrawn from the study – two for protocol deviations, and one who failed to attend further following recruitment. The 40 patients had a mean age of 32.4 years and 10 were male; the mean age of the control group was 34.4 years and six were male. Three of the 40 patients were lost to the study before the collection of the second plaque sample – one became pregnant, one was prescribed antibiotics, and one failed to attend for the second sample collection. Therefore, 117 plaque samples were available for sequence analysis on an intent-to-treat basis. No adverse events were reported by any of the study participants.

A total of 773,547 sequences were available for analysis after quality filtering. Of these, 3,845 sequences were randomly subsampled from each sample for the quantitative analysis. The α-diversity of the samples is shown in Table 1. There were no significant differences in the richness or diversity of the samples (Kruskal–Wallis test).

**Table 1.** Richness and diversity of plaque samples from different subject and treatment groups.

Treatment group	n	Mean no. of OTUs (SD)	Inverse Shannon index (SD)
Patients, gum base, baseline	20	405.1 (76.5)	37.8 (17.0)
Patients, gum base, 2 weeks	19	410.4 (94.4)	35.4 (15.8)
Patients, maltitol, baseline	20	379.8 (45.7)	31.0 (14.7)
Patients, maltitol, 2 weeks	18	386.6 (74.4)	32.3 (16.9)
Controls, gum base, baseline	20	359.9 (64.5)	31.2 (15.4)
Controls, gum base, 2 weeks	20	359.9 (62.0)	34.0 (13.9)

**Figure 1.**
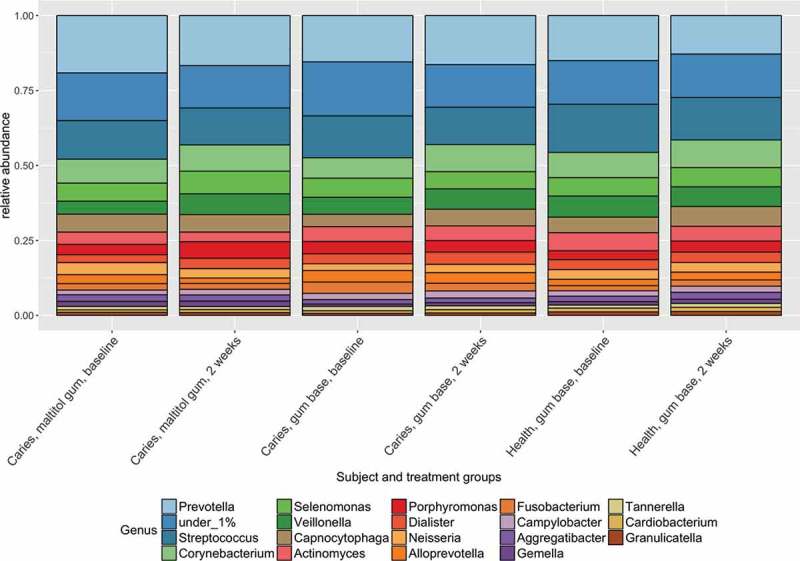
Distribution of predominant bacterial genera among groups and treatments.

Sequences were identified with reference to HOMD version 13.2. The composition of the plaque communities at genus level is shown in Figure 1 and was consistent with previous studies of the oral microbiome. Firmicutes, Bacteroidetes and Actinobacteria were predominant at phylum level and the most frequently detected genera were *Prevotella, Streptococcus, Corynebacterium* and *Veillonella*.

The membership (the species-level OTUs present in the sample, assessed by the J-class statistic) and structure (the OTUs present and their relative abundances, calculated using the θ-YC algorithm) of the communities in the different subject and treatment samples were compared. There were no differences in either the structure or membership of the sample communities, as compared by parsimony, before and after any of the treatments, nor between the patient and control groups either before or after treatment. HOMOVA comparisons of structure and membership also showed no differences between groups.

Similarly, comparison of the structure of the communities by AMOVA showed no differences between any of the groups. There was however a significant difference in community membership between the treatment groups, assessed by AMOVA (p = 0.009; experiment significance level p = 0.04; for pairwise comparisons, the Bonferroni-corrected value of p = 0.003 was used). There were no significant differences within groups before and after treatment. There was a significant difference between the control group after treatment and the maltitol patient group after treatment (p < 0.001).

**Figure 2.**
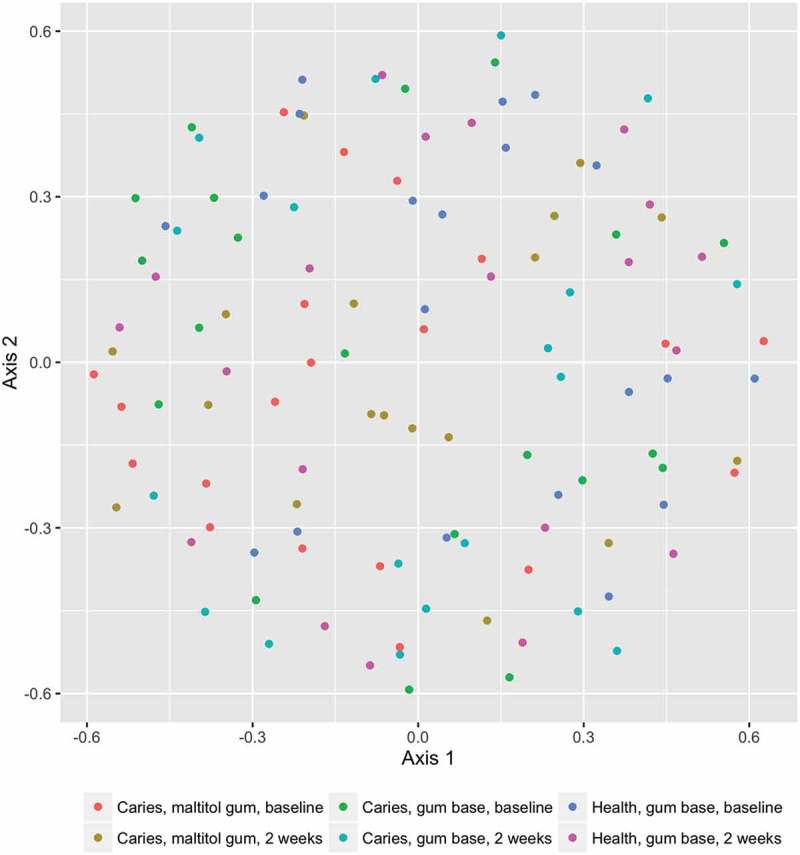
Non-metric multidimensional scaling (NMDS) plot comparing the bacterial community membership (J-class distances) of plaque samples before and after using chewing gum for 2 weeks.

**Figure 3.**
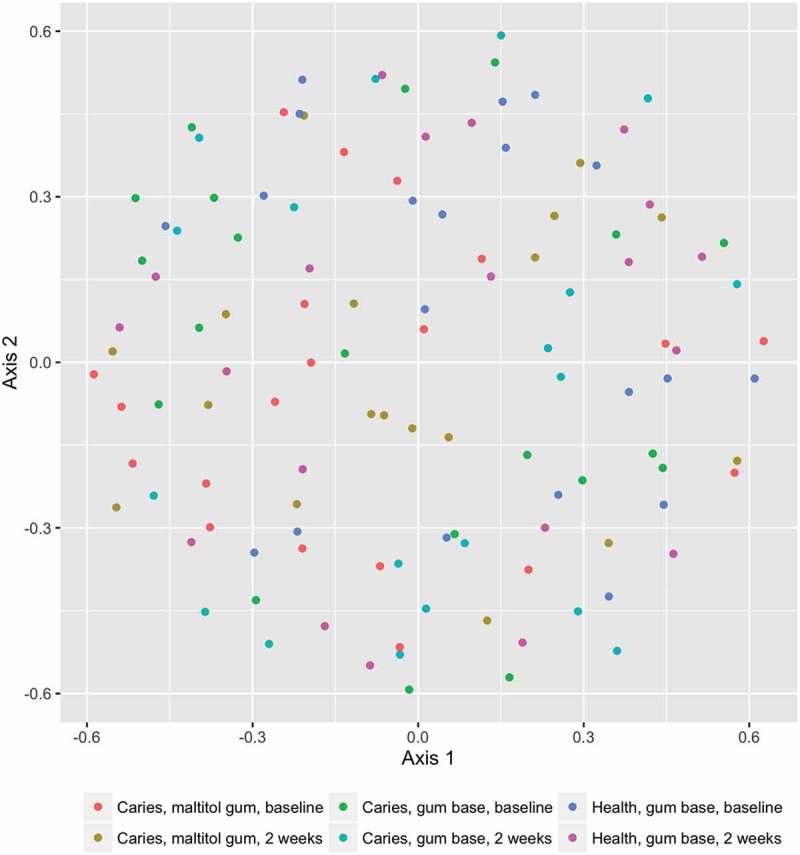
Non-metric multidimensional scaling (NMDS) plot comparing the bacterial community structure (θ-YC) of plaque samples before and after using chewing gum for 2 weeks.

Ordination plots based on NMDS analysis of the J-class and θ-YC data are shown in Figures 2 and 3. Figures 2 and 3 confirm the statistical analysis: it can be seen that the sample microbial communities in the different treatment groups completely overlap, showing that the treatments did not deeply impact the community composition.

**Figure 4.**
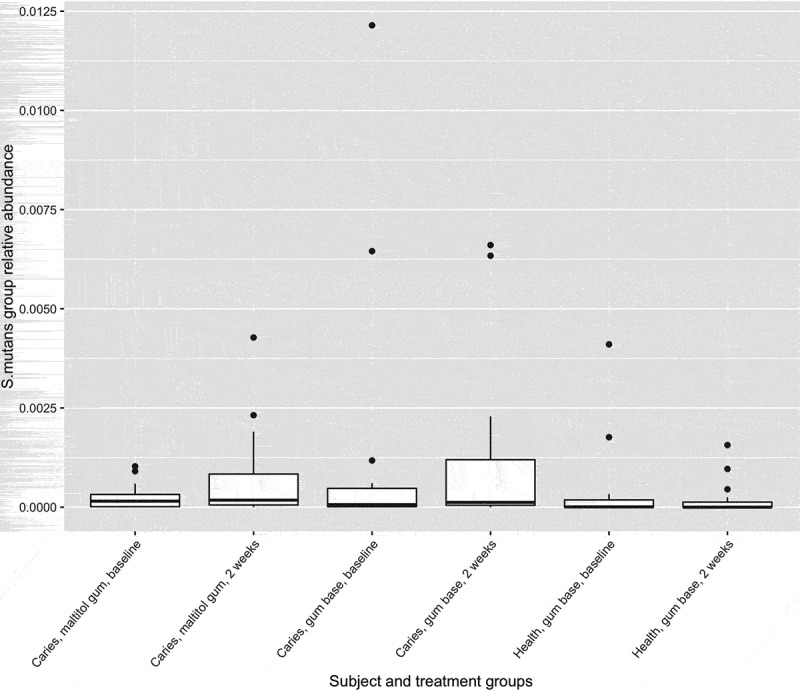
Box plot showing mutans-group streptococci as a proportion of the total microbiota. Upper and lower edges of the boxes are the first and third quartiles; the line inside the box is the second quartile (median); individual dots are outliers.

**Figure 5.**
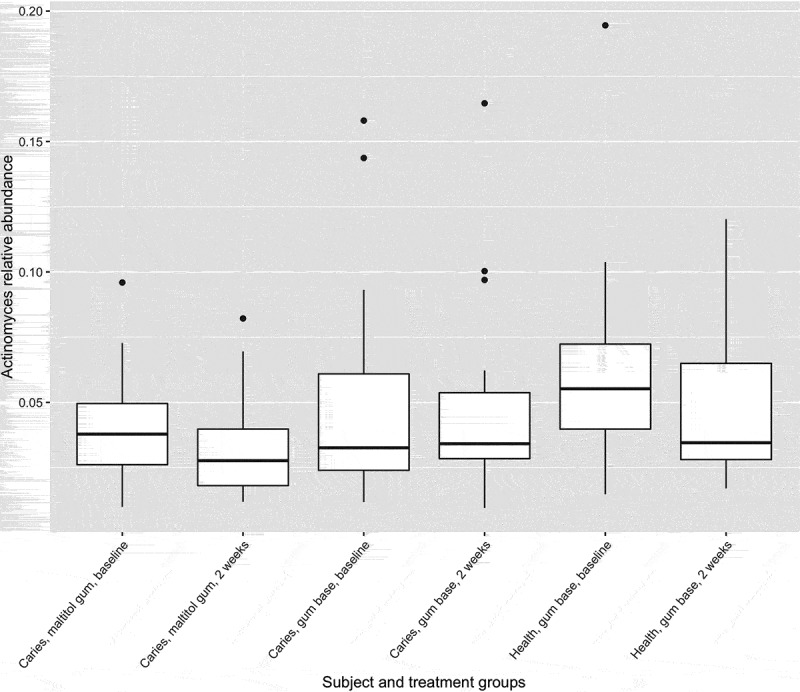
Box plot showing *Actinomyces* as a proportion of the total microbiota. Upper and lower edges of the boxes are the first and third quartiles; the line inside the box is the second quartile (median); individual dots are outliers.

**Figure 6.**
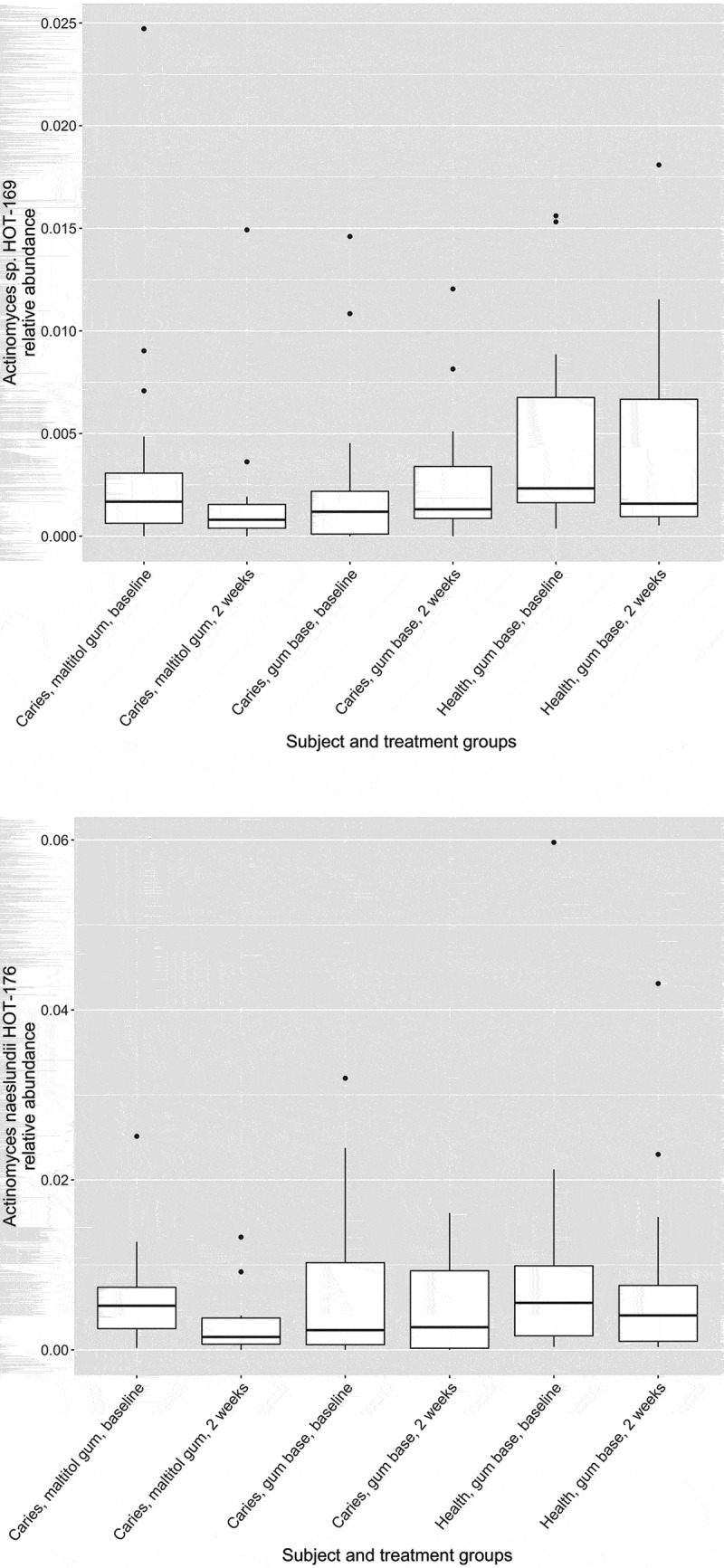
Box plots showing proportions of summed oligotypes in study treatment groups corresponding to *Actinomyces* species HOT_169 and HOT_176.

The proportions of sequences identified as belonging to the genus *Lactobacillus* and mutans-group streptococci (proportions of *Streptococcus mutans* and *Streptococcus sobrinus* combined; see Figure 4) were low in all treatment groups. Relative abundances of these organisms were compared within treatment groups by means of the Wilcoxon signed-rank test, which compares paired data. For *Lactobacillus*, there were no differences before and after treatment for either patient group but relative abundance was significantly reduced after treatment in the control group. For mutans-group streptococci, there were no differences before and after treatment for the control group and the patient group treated with the gum base, but relative abundance was significantly increased in the patient group who used the maltitol gum.

The relative abundance of the genus *Actinomyces* in subject and treatment groups is shown in Figure 5. At baseline, the proportion of *Actinomyces* was significantly higher in healthy subjects than in patients with caries (p < 0.05, two-sample Wilcoxon test). There were no differences before and after treatment for any group however (paired Wilcoxon test). Of 33,777 *Actinomyces* sequences analysed by minimum entropy decomposition, 6,458 were removed as outliers due to the stringency of the parameters set, and 27,319 were binned into 262 MED nodes, equivalent to oligotypes. Oligotypes were identified by BLAST interrogation of the HOMD dataset and found to correspond to 26 species-level human oral taxa. Counts of oligotypes were combined to give species-level relative abundances, which were compared before and after treatment using the paired Wilcoxon test. Proportions of *A. naeslundii* HOT_176 (p = 0.0012) and *Actinomyces* HOT_169 (p = 0.0008) were significantly reduced following use of maltitol chewing gum in the patients (significance threshold following Bonferroni correction: 0.05/26 = 0.0019) (Figure 6).

## Discussion

This study has shown that the regular consumption of chewing gums with or without maltitol had only minor effects on the composition of the dental plaque microbiota as a whole in subjects with active caries; nor was there any effect with gum base alone in control subjects without caries. This finding is not unexpected, as it is known that the human diet has little influence on the composition of the oral microbiome. Individuals on omnivorous, ovo-lacto-vegetarian, or vegan diets showed no differences in the composition of their salivary microbiota [15]. Furthermore, few differences have been observed in the oral microbiome of individuals residing in different geographical locations with differing typical diets [16]. Placing food in the mouth and chewing stimulate salivary flow, and the food is swallowed before oral bacteria can use it as substrate. The primary exception to this is simple sugars, which, because they are part of the normal degradation pathway of salivary glycoproteins, can be rapidly taken up by bacteria and fermented to form acid [17,18].

The subjects used the chewing gum for only 2 weeks, and it is possible that extended use of the maltitol-supplemented gum would be needed to show an effect, given that the resilience of the oral microbiome is well known. The results obtained in this study were similar to those reported for another sugar alcohol, xylitol, where it was shown that use of xylitol chewing gum for 5 weeks had no effect on the composition of the salivary microbiota, although levels of *S. mutans* were reduced after using both xylitol-containing and control chewing gum [19]. A longer duration of treatment may therefore have shown an effect of chewing on levels of bacterial caries markers, and should be investigated for effects on plaque microbiota composition.

The levels of the caries-associated bacterial groups, mutans-group streptococci, and lactobacilli in plaque were very low, even in the caries-active group. This confirms that the differences that have been seen in the numbers of these bacteria in caries have been highlighted by the use of selective media that can detect very low levels of the organisms [20]. The role of these bacteria in the disease process has been questioned, because they constitute such a minor component of the microbiota, even in individuals with caries; and it is now thought that the plaque biofilm as a whole is responsible for the early stages of the disease [1]. Because the levels of mutans-group streptococci and lactobacilli were so low in this study, the findings that lactobacilli were reduced in the control group and mutans-group streptococci raised in the patient group receiving maltitol should be interpreted with caution.

Some differences were seen in the proportions of specific *Actinomyces* taxa after use of the maltitol gum. These taxa were all present at very low levels in the plaque biofilm, so the clinical significance of this finding is unclear. *Actinomyces* species are known to be acidogenic and aciduric, and have been implicated in caries [21], particularly that of root surfaces [22], although the relative abundance of the genus in this study was lower in patients with caries than in controls. They also play an important role in plaque formation itself, being among the primary colonisers of tooth surfaces [23,24].
